# Genetic Mutations and Non-Coding RNA-Based Epigenetic Alterations Mediating the Warburg Effect in Colorectal Carcinogenesis

**DOI:** 10.3390/biology10090847

**Published:** 2021-08-30

**Authors:** Batoul Abi Zamer, Wafaa Abumustafa, Mawieh Hamad, Azzam A. Maghazachi, Jibran Sualeh Muhammad

**Affiliations:** 1 Department of Basic Medical Sciences, College of Medicine, University of Sharjah, Sharjah 27272, United Arab Emirates; batoul.abizamer@gmail.com (B.A.Z.); u20104813@sharjah.ac.ae (W.A.); 2 Sharjah Institute for Medical Research, University of Sharjah, Sharjah 27272, United Arab Emirates; mabdelhaq@sharjah.ac.ae (M.H.); amagazachi@sharjah.ac.ae (A.A.M.); 3 Department of Medical Laboratory Sciences, College of Health Sciences, University of Sharjah, Sharjah 27272, United Arab Emirates; 4 Department of Clinical Sciences, College of Medicine, University of Sharjah, Sharjah 27272, United Arab Emirates

**Keywords:** colorectal cancer, Warburg effect, epigenetic alterations, ncRNA, genetic mutations, anti-glycolysis therapy

## Abstract

**Simple Summary:**

Colorectal cancer is one of the most leading causes of death worldwide. The Hallmark of colorectal cancer is the increase of glucose uptake and lactate production even in the presence of oxygen, a phenomenon known as the “Warburg effect”. This review summarizes the genetic mutations and epigenetic alterations, focusing on non-coding RNA associated with the oncogenes, tumor suppresser genes, and enzymes involved in the “Warburg effect”, in addition to their clinical impacts on colorectal cancer. This knowledge may open the door for novel therapeutic approaches to target colorectal cancer.

**Abstract:**

Colorectal cancer (CRC) development is a gradual process defined by the accumulation of numerous genetic mutations and epigenetic alterations leading to the adenoma-carcinoma sequence. Despite significant advances in the diagnosis and treatment of CRC, it continues to be a leading cause of cancer-related deaths worldwide. Even in the presence of oxygen, CRC cells bypass oxidative phosphorylation to produce metabolites that enable them to proliferate and survive—a phenomenon known as the “Warburg effect”. Understanding the complex glucose metabolism in CRC cells may support the development of new diagnostic and therapeutic approaches. Here we discuss the most recent findings on genetic mutations and epigenetic modulations that may positively or negatively regulate the Warburg effect in CRC cells. We focus on the non-coding RNA (ncRNA)-based epigenetics, and we present a perspective on the therapeutic relevance of critical molecules and ncRNAs mediating the Warburg effect in CRC cells. All the relevant studies were identified and assessed according to the genes and enzymes mediating the Warburg effect. The findings summarized in this review should provide a better understanding of the relevance of genetic mutations and the ncRNA-based epigenetic alterations to CRC pathogenesis to help overcome chemoresistance.

## 1. Introduction

Colorectal cancer (CRC) is the fourth most common type of cancer, with more than 1.84 million new cases reported annually. Despite improvements in diagnosis and treatment, CRC causes approximately one million deaths per year and accounts for the third-highest cancer-related deaths worldwide [1]. Recently, anti-cancer drugs targeting dysregulated cancer cell metabolism have been gaining greater attention in the scientific community [2,3]; therefore, understanding the metabolic pathways in CRC cells may provide a key for developing novel diagnostic and therapeutic options to overcome this disease.

Glucose is metabolized by oxidative phosphorylation (OXPHOS) when oxygen is available to normal cells. Under hypoxic conditions, cells undergo anaerobic glycolysis to produce lactate [4], but even in the presence of oxygen, cancer cells adapt to metabolize a high amount of glucose into lactate to fuel uncontrolled cell growth. This phenomenon is the “Warburg effect”, which is a hallmark of nearly all types of cancer, including CRC [5,6]. CRC is a heterogeneous disease in which numerous oncogenes and tumor suppresser genes are mutated in an adenoma-carcinoma sequence that facilitates CRC progression [1]. Several genetic mutations may lead to the upregulation of enzymes and transporters involved in the Warburg effect. Indeed, CRC cells prefer glycolysis over OXPHOS even under normoxic conditions, leading to mitochondrial dysfunction [7]. Still, it is unclear whether OXPHOS is completely turned off by genetic and epigenetic alteration or whether mitochondria continue to function to generate the energy required by the CRC cells. The former might be true because the net ATP yield generated by OXPHOS is higher than that generated by aerobic glycolysis, but glycolysis is 100 times faster [8]. Moreover, glycolysis protects CRC cells against the toxic byproducts of OXPHOS and provides an acidic environment to enhance the cellular uptake of the essential intermediate metabolites required for proper cancer cell growth [9]. Regardless of the significance of the Warburg effect, some types of cancer depend on more than 90% OXPHOS [8], but some cancer metabolisms are a mixture of OXPHOS and glycolysis [9]. Kaldma et al. reported that in situ human CRC cells use glycolysis in the same way as healthy cells; nevertheless, in malignant cells, increased OXPHOS might be due to stimulation of the mitochondrial biogenesis [10].

Researchers have tried to understand whether OXPHOS is the primary source of energy in all stages of CRC. They have noted gradual metabolic changes in the adenoma-carcinoma sequence in CRC cells [11], which might result from a shift from OXPHOS to glycolysis and the accumulation of mitochondrial DNA mutations during the development of CRC [11]. Furthermore, tumor suppressor expression, which negatively regulates glycolysis, has also been shown to decline during adenoma-carcinoma progression [12]. CRC cells carrying *KRAS* mutations had higher mitochondrial activity than *BRAF* mutations and showed higher glycolytic activity [13]; thus, *KRAS* and *BRAF* should be considered prognostic markers of OXPHOS or glycolysis in CRC patients. However, another study found that mitochondrial activity and OXPHOS were higher in normal cells surrounding CRC cells, which was inconsistent with the “*reverse Warburg effect*” [8]. Altogether, this provides a better understanding of the reason behind the heterogeneity of CRC metabolism. Understanding the mechanisms by which tumor cells generate energy and the exact role of the microenvironment are matters of interest for drug design and CRC treatment.

Researchers have shown that CRC tumor metabolism is mainly reprogrammed by the accumulation of epigenetic alterations [14], which are heritable phenotypic changes in gene expression that do not alter the DNA sequence but potentially lead to cancer development. These epigenetic alterations mainly involve DNA methylation, histone modification, and the processes mediated by non-coding RNAs (ncRNAs), including microRNAs (miRNAs) and long non-coding RNAs (lncRNAs) [15]. Interestingly, it has been found that ~98% of the non-protein-coding genome known as ncRNA plays a role in regulating the gene expression of several genes under both physiological and pathological conditions, such as CRC [16]. Those genes could be involved in various key cellular processes, including invasion and metastasis, cell proliferation, differentiation, and metabolism. Thus, these ncRNA are categorized as oncogenes and tumor suppresser genes. One of the most studied ncRNA in CRC is the HOX transcript antisense intergenic RNA (HOTAIR), which is a lncRNA that binds in trans with polycomb repressive complex 2 (PRC2) and increases the expression of several genes involved in invasion and metastasis of CRC, such as vimentin and E-cadherin [16]. Other ncRNAs play a critical role in rewiring CRC cell metabolism by altering the activity of vital metabolic enzymes [17]. Since alterations at the epigenetic level are generally reversible, studying them may provide novel targets for anti-cancer therapy.

This review summarizes the current knowledge about genetic mutations and epigenetic alterations, focusing on the functional role of ncRNA-mediated regulation of the Warburg effect in CRC. Also, we summarize novel therapeutic drugs targeting the critical molecules involved in CRC cell metabolism and the Warburg effect.

## 2. Materials and Methods

**Figure 1:**
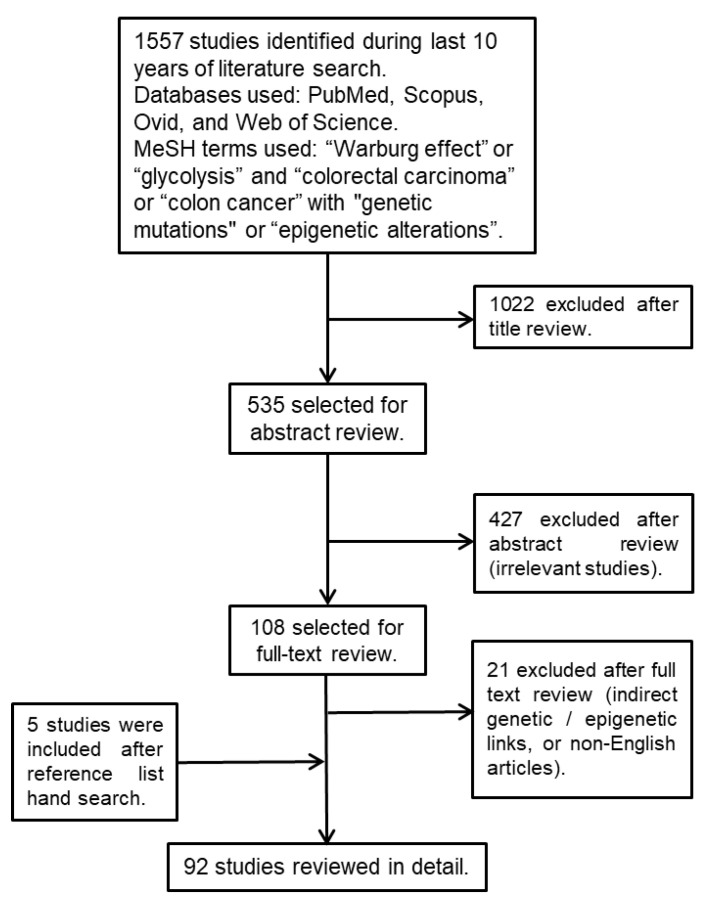
Flowchart outlining the strategy employed to identify the relevant studies.

We used several medical subject heading (MeSH) terms, such as “Warburg effect” or “glycolysis” and “colorectal cancer” or “colon/rectal cancer” with “genetic mutations” or “epigenetic alterations”, to search the SCOPUS, Ovid, PubMed, and Web of Science scientific databases for studies published during the past ten years. To identify additional studies, we manually searched the reference lists of the selected studies (Figure 1).

## 3. Metabolic Reprogramming in CRC: Genetic Mutations and ncRNA-Mediated Epigenetic Alteration

Recent studies have suggested that numerous genetic mutations and epigenetic alterations causing abnormal activation of several oncogenes (*KRAS* [18,19], *c-Myc* [20], *PIM1* [21]), and the inactivation of several tumor suppresser genes (*APC* [22], *TP53* [23], *SMAD4* [24], *PTEN* [25]), reprogram the metabolic pathway in CRC, mediating the Warburg effect. For instance, *KRAS* expression can be downregulated by the overexpression of miR-143, while the lncRNA (glycolysis-associated lncRNA of colorectal cancer) GLCC1 directly increases *c-Myc* expression [16,26]. In addition, miR-135, miR-150-5p, miR-34a, and miR-21 target *APC*, *TP53*, *SMAD4*, and *PTEN* expression to promote CRC progression [16,27]. Also, aberrant activation of various signaling pathways, such as the Wnt/β-catenin, FYN-HIF2A, Receptor Tyrosine Kinase (RTK)/Ras GTPase/MAP kinase (MAPK), and PI3K pathways, modulates CRC cell metabolism [28]. Activation of the Wnt/β-catenin pathway accounts for almost 90% of sporadic CRC and is usually associated with a high rate of aerobic glycolysis [22,29]. Also, the Hippo pathway induces glycolysis via upregulation of yes-associated protein 1 [30]. Mutations in transcription factors such as forkhead box (*FOX*) [6] and *HIF1A* [5] genes, which could alter the expression of the enzyme-coding genes involved in glycolysis, have been widely reported in CRC. Those enzymes include phosphoglycerate kinase 1 (PGK1) [31], glucose transporter 1 (GLUT1), hexokinase 2 (HK2), pyruvate kinase M2 (PKM2), and lactate dehydrogenase (LDH) [29]. Furthermore, the cytokine-mediated pro-inflammatory microenvironment may enhance the Warburg effect in CRC [32].

Cancer cells have altered epigenetic mechanisms to manipulate the gene expression for abnormal cell growth and metastasis [33]. These epigenetic mechanisms include DNA methylations, histone modifications, and ncRNA-mediated regulation of gene expression [34,35]. Regulation of gene expression by ncRNAs can happen at multiple levels by interacting with DNA, RNA, or proteins. Moreover, the ncRNAs can modulate chromatin structure and the transcription of adjacent or faraway genes. However, the best of the known mechanisms of ncRNA-mediated gene repression is interference with the transcription machinery, which leads to alteration of the recruitment of transcription factors. The ncRNAs mediate transcriptional regulation on an epigenetic level through interaction with chromatin modifiers, either directly via chromatin looping or by sponging a diversity of miRNAs [36]. Recently, accumulating evidence has also proven the role of regulatory ncRNA in metabolic remodeling of CRC [37,38,39]. These molecules can either function as tumor inducers or suppressers by targeting different players of CRC metabolism genes such as transcription factors, enzymes, and transporters of glycolysis. Profiling those ncRNAs in CRC metabolism is further required, paving the way to identify novel targeted therapies and diagnostic, prognostic, and predictive tests. Nevertheless, ncRNAs are the most studied cause of epigenetic-based metabolic reprogramming in CRC, and are hereafter discussed in more detail.

### 3.1. Oncogenes and Tumor Suppressor Genes

CRC usually occurs as a result of accumulating genetic mutations or epigenetic alterations. Several pathway models have been proposed for CRC development in the adenoma-carcinoma sequence [40], including chromosomal instability, accounting for almost 85% of CRC cases. Karyotypic changes resulting from the loss or gain of chromosomal segments, and chromosomal rearrangement of the loss of heterozygosity (LOH), are common alterations in this model [40]. Consequently, oncogenes such as *KRAS*, *BRAF*, and *c-Myc* are upregulated, and tumor suppresser genes such as *APC* and *TP53* are inactivated, leading to cancer. Also, the LOH of chromosome 18q causes the deletion of tumor suppresser genes such as *DCC*, *SMAD2*, and *SMAD4*, the mutation of which leads to the upregulation of enzymes involved in the glycolysis pathway, such as PKM2, HK2, and GLUT1 [29], as an early event in premalignant colonic mucosa and CRC progression [41].

#### 3.1.1. *KRAS* Proto-Oncogene

The *KRAS* gene is a member of the *RAS* family of oncogenes and the well-studied superfamily of small GTPases in cancers [42]. *KRAS* mutations account for more than one-third of CRC cases [43], and this high frequency is evidence of their significant role in the initiation and progression of CRC. Current knowledge has shed light on the importance of *KRAS* in cancer metabolism, which is mainly achieved when mutated *KRAS* enhances the transport of glucose intermediates into different pathways, such as the pentose phosphate pathway and the hexosamine biosynthesis pathway [44]. Furthermore, upregulated expression of *KRAS* leads to abnormal phosphorylation of the kinases in the downstream Mitogen-Activated Protein Kinase/Extracellular Signal-Regulated Kinase (MAPK/ERK) signaling pathway. When the kinases in this pathway are phosphorylated, a group of genes and transcription factors that play a role in cell proliferation, survival, and glucose metabolism are activated. Glucose metabolism can be measured by the accumulation of fluorine-18 deoxyglucose (18F-FDG) in cancer cells. One study found that *KRAS* mutant CRC cells had greater 18F-FDG accumulation than wild-type ones. The increase in glucose uptake was observed to be strongly associated with *GLUT1* and *HIF1A* mutations. GLUT1 is one of the most important enzymes upregulated in CRC and is associated with the *KRAS* mutation which enhances glucose absorption by cancer cells [18].

A recently raised question is whether resistance to anti-Epidermal Growth Factor Receptor (EGFR) therapy, which is associated with the *KRAS* mutation, is caused by metabolic reprogramming in CRC or is a separate event. EGFR treatment’s relationship with glycolysis has been established in different cancers, including bladder, glioma, breast, and head and neck cancers [45,46,47]. However, this relationship was poorly understood in CRC until Ye et al. reported that AMP-activated protein kinase (AMPK) was the key player in this relationship [47]. An increase in glycolysis caused the inhibition of AMPK phosphorylation and, thus, impaired anti-EGFR therapy for treating CRC cells. This conclusion followed the treatment of *KRAS* mutant CRC cells with a glycolysis inhibitor, which caused restoration of AMPK phosphorylation and anti-EGFR sensitization in CRC cells. Additionally, the cause of resistance to cetuximab (an anti-EGFR antibody) in CRC cells with mutant *KRAS* was due to an accumulation of methylglyoxal (MGO)—a byproduct of glycolysis—and activation of the AKT enzyme [48]. CRC cells under MGO-mediated stress exhibited AKT activation and resistance to anti-EGFR therapy. MGO scavengers downregulated AKT activity and restored the sensitivity of *KRAS* mutant CRC to cetuximab [48]. In light of these studies, no novel potential glycolysis-related targets have been shown to improve responses to anti-EGFR therapy in *KRAS* mutant CRC.

Epigenetic alteration of *KRAS* has been observed to modulate different cellular pathways and delay CRC progression [39]. However, the epigenetic alteration of *KRAS* that affects its role in glycolysis is still unclear. One study has shown that the knockdown of UNC5B antisense lncRNA 1 (UNC5B-AS1) significantly inhibits the glycolysis of pancreatic ductal adenocarcinoma cells carrying *KRAS* mutations [49]. The same lncRNA exhibited an oncogenic activity in CRC. Another study reported that UNC5B-AS1 silencing was associated with rising miR-622 levels. Altogether, these studies may provide novel insights into the role of UNC5B-AS1 in increasing *KRAS* activity and promoting glycolysis in CRC cells [50].

Although *KRAS* promotes resistance to anti-EGFR antibodies, it enhances responses to different glycolysis inhibitors such as 3-bromopyruvate (3-BrPA) and ascorbic acid (AA) treatment [18,51]. *KRAS* mutation allows the stable shift of CRC cells toward glycolysis; thus, it has been proposed that CRC cells carrying *KRAS* mutations exhibit greater sensitivity to 3-BrPA than CRC cells lacking *KRAS* mutations [51]. Furthermore, another study reported an improved therapeutic response to AA in CRC patients with *KRAS* mutations, inhibiting *KRAS* phosphorylation activity and promoting *KRAS* detachment from the cell membrane and inhibiting various downstream enzymes involved in glycolysis [18]. These treatments have proved that glycolytic inhibitor activity improves with the upregulation of particular oncogenes, such as *KRAS*, involved in glycolysis. Despite the design of different strategies to modulate deregulated *KRAS* activity, they have failed to reach the clinical trial stage, especially in terms of resistance to chemotherapy [19]. These observations provide evidence for targeting the metabolic oncogenic activity of *KRAS* in sensitizing CRC cells to different chemotherapies.

#### 3.1.2. *c-Myc* Proto-Oncogene

Previously, almost 70% of CRC cases were reported to have *c-Myc* mutations, and they were considered to be an important event during CRC development [52]. Although our search of the cBioPortal database indicated a much lower incidence of *c-Myc* mutations (around 5%), *c-Myc* might still play a significant role in CRC (www.cbiportal.com, accessed on 25 June 2021). *c-Myc* is a transcription factor that regulates many genes involved in protein biosynthesis, cellular proliferation, and metabolism. Mutations in *c-Myc* have been extensively linked to glycolysis in cancer [53]. Specifically, metabolic reprogramming in CRC is associated with aberrant *c-Myc* activation, which plays a pivotal role in manipulating 121 metabolic genes [53]; for example, it downregulates mitochondrial biogenesis genes but upregulates genes relating to DNA and histone modification. Interestingly, *c-Myc* has been reported to facilitate the transcription of almost all the genes responsible for aerobic glycolysis [54]. 

Mutations cause aberrant expression of *c-Myc* via different pathways, such as the Wnt and PI3K/AKT/mTOR signaling pathways [53]. When *c-Myc* is inhibited, various glycolysis enzymes, such as PDK1, LDH, and GLUT1, are downregulated, resulting in tumor growth suppression [20]. The upregulation of *c-Myc* indirectly promotes glycolysis in CRC cells through the overexpression of polypyrimidine tract binding protein 1 (PTB1)—a protein that plays a role in pre-mRNA splicing. PTB1 facilitates glycolysis by promoting the splicing of PKM2 in CRC cells [55]. In addition, *c-Myc* can also facilitate CRC progression by upregulating genes relating to other metabolic pathways. For instance, *c-Myc* has been shown to tip the balance of CRC metabolism from glycolysis toward OXPHOS by upregulating mitochondrial-related proteins such as PGC-1, CPT1A, and TFAM [56]. *c-Myc* boosts CRC progression through various mechanisms, such as OXPHOS and glycolysis, allowing the identification of novel molecules in its activity. Unfortunately, targeting *c-Myc* through small molecules such as antibodies is difficult because it is a nuclear protein without a deep surface binding pocket [53]. Therefore, further studies are required to identify other target therapies downstream of *c-Myc*, or examine different epigenetic mechanisms that modulate its expression in CRC cells.

*c-Myc* regulation can depend on epigenetics, including lncRNA and miRNA, to control the Warburg effect in CRC cells. The lncRNA MEG3 plays a critical role in glycolysis in CRC cells. MEG3 expression has been significantly associated with the progression of CRC. In light of this finding, its effect on *c-Myc* was examined, demonstrating that MEG3 downregulates *c-Myc* activity by promoting *c-Myc* degradation through ubiquitination and therefore inhibits glycolysis in CRC [57]. Unlike MEG3, another novel lncRNA GLCC1 was shown to protect *c-Myc* from degradation through ubiquitination, and this oncogenic lncRNA was associated with poor CRC prognoses [26]. The upregulation of *c-Myc* by GLCC1 enhanced the glycolysis of CRC. Inhibition of *c-Myc* ubiquitination is a mechanism used by oncomirs such as miR-181d. MiR-181d upregulates *c-Myc* expression to promote glucose uptake and lactate production, and facilitate CRC invasion and progression [58]. Furthermore, anti-oncomirs have exhibited a regulatory effect on *c-Myc*. A good example is miR-124, which downregulates *c-Myc* activity indirectly through the DDX6 gene to inhibit aerobic glycolysis of CRC [55]. This gene is a key molecule in the Warburg effect in CRC. Hence, understanding the epigenetics of *c-Myc* would provide a clear picture of its role in CRC development and aid in treating CRC.

Different anti-cancer therapies target *c-Myc* to inhibit CRC progression and metastasis. One of these treatments involves vitamin D and its receptor (VDR) [57], which have been reported to promote the upregulation of MEG3 and inhibit *c-Myc* activity. In addition, a body of evidence has proved that Ketamine (a pain reliever given to cancer patients) plays a role in delaying cancer progression. However, the mechanism underpinning its anti-tumor activity was unclear until Hu et al. reported that Ketamine might inhibit glycolysis in CRC cells [59], mainly through the inhibition of the N-methyl-D-aspartate receptor (NMDAR). When NMDAR is inhibited, *c-Myc* expression is downregulated, resulting in the attenuation of CRC cell viability and migration. Another anti-cancer drug that directly targets *c-Myc* expression is the Chinese medicine Dioscin. Recently, Dioscin was found to destabilize *c-Myc* by promoting its ubiquitination [60]. It is now clear that many anti-cancer treatments with uncertain activity might be linked to transcription factors involved in glycolysis in CRC cells, including *c-Myc*. Therefore, more studies are needed to understand their mechanisms, improve their activity, and potentially lead to other treatments with fewer adverse effects.

#### 3.1.3. Hypoxia-Inducible Factor 1-Alpha (HIF1-α)

Another example of a transcription factor with a role in remodeling CRC metabolism is HIF1-α [5]. As a tumor grows, it exceeds the limit of the blood supplied, leading to the tumor environment being less oxygenated than the normal environment from which the tumor emerged. This shortage of oxygen creates hypoxia, and transcription factors activated under such conditions are known as hypoxia-inducible transcription factors (HIFs) [61]. HIF1-α is a major example of a hypoxia-inducible transcription factor that promotes the activation of a group of genes by binding to their promoters. Simultaneously, these activated genes reprogram the glucose uptake of cancer cells. HIF1-α has been reported to upregulate the expression of key enzyme-coding genes involved in the Warburg effect while downregulating the genes involved in OXPHOS [20]. Additionally, HIF1-α mediates the Warburg effect in CRC by upregulating miRNAs such as LINC00511 [62]. Protein Proviral Integration of Moloney virus 2 (Pim-2) is a cofactor of HIF1-α and facilitates its function of promoting glycolysis in hypoxic conditions [63]. This highlights a new pathway for targeting the effect of HIF1-α in the progression of CRC through the Warburg effect. 

In addition to cancer progression, HIF1-α plays an important role in inflammatory diseases [64]. The effects of different anti-inflammatory chemotherapies have been tested on HIF1-α. One of the major naturally occurring anti-inflammatory and anti-cancer treatments is rosmarinic acid (RA). The effect of RA has been tested on CRC, and it was reported that RA had an anti-Warburg effect in CRC cells through the inhibition of HIF1-α. This was supported by the inhibition of glucose uptake and lactate production [65]. This finding provides support for considering HIF1-α as a good target for different chemotherapies to attenuate the Warburg effect in CRC. However, there are still concerns about the role of HIF1-α in resistance to chemotherapy. The regulatory mechanism is by the factor inhibiting HIF-1 (FIH-1) binding to and inhibiting HIF1-α. Nakagawa et al. found that epigenetic downregulation of *FIH-1* through miR-31 upregulated HIF1-α and glycolysis and promoted resistance to 5-fluorouracil (5-FU) [66]. Understanding the mechanism by which HIF1-α induces drug resistance through glycolysis is important for improving the responses of CRC cells to different chemotherapies.

Epigenetic alteration of *HIF1A* has recently been found to occur at the post-transcriptional level [67]. Unlike chromatin-modifying proteins that target DNA, histones, or chromatins, RNA-modifying proteins (RMPs) modify RNA. RMPs are divided into three groups. Chemical marks are deposited by enzymes called ‘writers’ and eliminated by ‘erasers’. These chemical modifications are usually recognized by ‘readers’. HIF1-α was found to be repressed when N6-methyladenosine (M6A) modification of the writer RMP methyltransferase-like3 (*METTL3*) was knocked down in CRC cells [67]. M6A (the most prevalent mRNA modification) is methylation that occurs in the N6-position of adenosine [68]; hence, this knockdown was caused by glycolysis repression. Meanwhile, the overexpression of the reader YTHDF1 rescued the inhibition of HIF1-α and upregulated the Warburg effect. Epigenetic-mediated positive regulation of HIF1-α-induced glycolysis through lncRNA has also been reported [38], but in cancers other than CRC. The HIF1-α antisense lncRNA (HIFAL) has been found to specifically upregulate HIF1-α activity in the glycolysis of breast cancer; however, further studies that focus on CRC are warranted [38]. Furthermore, HIF-1α has been found to be a direct target for histone demethylation by the histone demethylase JMJD2D. JMJD2D upregulates *HIF1A* expression by demethylating H3K9m2/3 on its promoter region. JMJD2D and SRY-Box Transcription Factor 9 (SOX9) upregulate the mechanistic target of rapamycin (mTOR) to increase HIF-1α expression. Inhibition of JMJD2D in CRC cells reduces HIF-1α expression, glucose consumption, and lactate production [69]. These epigenetic alterations provide novel therapies for modulating CRC metabolism by targeting HIF-1α.

#### 3.1.4. Adenomatous Polyposis Coli (*APC*)

One of the main players in CRC is the tumor suppresser gene *APC*. Mutations in this gene are accompanied by the activation of the Wnt signaling pathway [22]. *APC* mutations may be involved in the metabolic reprogramming of CRC cells via activation of the Wnt signaling pathway [22]. When this pathway is activated, β-catenin accumulates, thereby upregulating the key player enzymes of the glycolytic pathway—PKM2, LDHA, and PDK1. 

Different anti-cancer treatments have been shown to target APC to inhibit glycolysis in CRC cells. Dwarf lilyturf tuber 13 (DT-13) is an anti-tumor drug with a significant effect on tumors and low toxicity for normal cells. However, the mechanism by which it works and its targets are still being tested. A recent study using *APC* deficient mice has suggested that DT-13 inhibits glycolysis and thus CRC progression [2]. DT-13 inhibited the aerobic glycolysis enzymes that were upregulated because of deregulated *APC*. Although the direct activity of DT-13 on APC was not tested in this study, it was still clear that APC might be another target for this drug. Moreover, treatment of CRC organoids with metformin—an anti-cancer drug—has shown a significant anti-cancer effect, especially in the early stages when *APC* mutations are reported [70]. Surprisingly, glycolysis activity was upregulated, suggesting that *APC* mutations might be involved in other pathways facilitating CRC progression. 

Epigenetics has been reported to either restore or inhibit the activity of APC. A biotinylated variant of *N*-((5-chloro-8-hydroxyquinoline-7-yl) (4-(diethylamino)phenyl)-methyl) butyramide (CBA-1) has been shown to epigenetically promote the upregulation of the Wnt pathway through its partner KDM3A [71]. The latter is a demethylase enzyme that promotes the demethylation of the transcription marker histone H3′s lysine 9 (H3K9Me2). Treating cells with CBA-1 significantly inhibits the Wnt signaling pathway and hence inhibits CRC cell growth with *APC* or β-catenin mutations and *APC* or *KRAS* mutations, providing evidence that APC might be a direct target for CBA-1.

#### 3.1.5. *TP53*

P53, encoded by the tumor suppresser gene *TP53*, regulates most important cellular processes, such as repairing damaged DNA, regulating cell arrest, senescence, apoptosis, and cellular metabolism, by regulating rate-limiting enzymes in glycolysis, including pyruvate dehydrogenase kinase 2 (PDK2) [23]. P53 downregulates *PDK2* through the upregulation of miR-149 when dichloro-acetate (DCA) is introduced into CRC cells. DCA is a drug mainly used to treat metabolic diseases, and recently, its effect was tested as an anti-cancer drug. Interestingly, when CRC cells were treated with DCA, aerobic glycolysis was inhibited, OXPHOS was upregulated, and sensitivity to chemotherapy was restored. Thus, besides playing a role in inhibiting glycolysis, P53 plays an important role in reversing resistance to chemotherapy. 

Restoring the expression of *TP53* can be achieved by different anti-cancer drugs such as FK866, which is an inhibitor of NAD metabolism. When FK866 was introduced into CRC cells, nicotinamide phosphoribosyltransferase (NAMPT) was inhibited, and consequently, Sirtuin 1 (SIRT1)—a histone deacetylase that deacetylates the lysine residue 382 of *TP53* to inhibit its activity—was reduced [72]. Consequently, *TP53* expression was restored, and CRC metabolism was disrupted. Studies involving the interaction between improved sensitivity to chemotherapy by restoring P53 activity and the reprogramming of CRC metabolism have recently gained greater attention. Restoration of P53 activity can be achieved by understanding the key genes and pathways of glycolysis regulated by P53 that could promote or prevent resistance to cancer therapies. 

### 3.2. Key Enzymes and Transporters Involved in the Warburg Effect

#### 3.2.1. Mitochondrial Pyruvate Carrier (MPC)

Accumulated evidence has demonstrated the important role of different enzymes and transporters in shifting OXPHOS toward glycolysis, and one of the key transporters is MPC. Recently, mutations causing the downregulation of MPC have been shown to enhance the Warburg effect [29]. MPC is an inner mitochondrial membrane protein that transports pyruvate to the mitochondria to mediate OXPHOS. MPC is generated by the expression of two genes, namely *MPC1* and *MPC2*. Recent studies have reported that *MPC1* is downregulated in CRC cells, and this downregulation is associated with an increased Warburg effect and CRC progression [73,74]. However, when *MPC1* was re-expressed, the growth properties of CRC cells were manipulated [75]. Moreover, aerobic glycolysis was inhibited in favor of oxidative metabolism [76]. Another study showed that *MPC1* inhibition was accompanied by upregulation of another metabolic pathway (known as glutamine metabolism), which mediates CRC progression [77]. Altogether, these studies provide insights into the actual purpose of *MPC1* in the metabolic network of CRC, thus aiding the design of advanced drugs to target the MPC axis. 

Studies about MPC regulation at the epigenetic level are lacking, but one study has reported that MPC activity is post-transcriptionally regulated in CRC. The post-transcriptional regulation of MPC was observed at two main acetylation sites: K45 and K46 [76]. The high glucose concentration upregulated the activity of the major mitochondria NAD^+^-dependent deacetylase Sirtuin 3 (SIRT3), which binds to and deacetylates *MPC1*, upregulating its inhibitory effect on CRC progression [76]. Studies on the epigenetic alteration of *MPC1* in CRC are still ongoing, and such studies are essential for identifying alternative pathways to target MPC in clinical cancer research.

#### 3.2.2. Pyruvate Kinase Isozyme M2 (PKM2)

PKM2 is a subtype of the pyruvate kinase family, along with L, R, and M1, which are tissue-specific isomers [78]. PKM1 and PKM2 result from a single *PKM* gene but with alternative splicing. Although PKM2 is expressed in almost all proliferating cells, especially cancer cells, PKM1 is mainly expressed in tissues with high energy demand, such as the brain, heart, and muscles. In fact, during embryonic development, PKM2 is gradually replaced with PKM1 [78]. However, in cancer cells, PKM2 expression is upregulated, causing the downregulation of PKM1. PKM2 in cells can be present as active tetramers or dimers with fewer active forms. PKM2 dimers are upregulated in the rate-limiting step of tumor cell glycolysis, promoting the switch from OXPHOS toward lactate production [78]. PKM2 activity has been shown to be upregulated by the Wnt/β-catenin and EGFR/MAPK signaling pathways, which are the major signaling pathways altered in CRC patients [22,79]. Notably, PKM2 dimers are translocated to the nuclei to work as cofactors of key transcriptional factors, such as HIF1-α, and promote glycolysis [80]. PKM2 activity has also been reported to be post-transcriptionally upregulated through its partner, Pim-2 [81]. 

The mRNA splicing of *PKM* to form PKM1 or PKM2 is mediated by the three heterogeneous nuclear ribonucleoproteins (hnRNPs)—PTB1, hnRNAPA1, and hnRNAPA2. Recently, studies have demonstrated that PKM2 levels can be epigenetically altered by targeting *PKM* splicers. Both miR-1 and miR-133b have been shown to inhibit CRC progression by targeting PTBP1. When inhibited, PTBP1 tipped the balance of PKM2 towards PKM1, thereby inhibiting glycolysis [82]. In addition, miR-124, miR-137, and miR-340 have been observed to reduce the growth of CRC cells by inhibiting PKM2 splicing, thereby counteracting the Warburg effect [83]. A newly discovered miR-206 has been shown to target hnRNPA1 and inhibit PKM2 splicing. The reduced PKM2 splicing inhibited glycolysis in CRC cells [84]. The lncRNA MEG3 has also been reported as a tumor suppressor in CRC cells by inhibiting *c-Myc* expression and indirectly reducing PKM2 activity, resulting in reduced glycolysis activity [57]. Another study showed that PKM2 levels were elevated by the lncRNA FEZF1-AS1, which improves PKM2 stabilization and thus enhances glycolysis in CRC cells [85]. Together, these studies prove that miRNAs and lncRNAs may impair CRC progression by targeting the Warburg effect and altering the PKM2/PKM1 ratio. 

PKM2 has been found to enhance the chemoresistance ability of CRC cells [84], based on a study in which PKM2 levels were significantly higher in oxaliplatin-resistant CRC cells than oxaliplatin-sensitive cells [86]. Moreover, resistance to chemotherapy was accompanied by elevated glycolysis activity. Interestingly, PKM2 was able to gradually eliminate the sensitivity of the nearby CRC cells to oxaliplatin. The circular RNA has-circ-0005963 (ciRS-122) was delivered to these cells through exosomal machinery and sponging miR-122—the main negative regulator of PKM2 activity. This study provided evidence that PKM2 may serve as a target for sensitizing CRC cells to different therapies [86]. 

Recently, butyrate from the dietary fiber was found to suppress CRC tumorigenesis in a mouse model [87]. This study also reported that dephosphorylation of PKM2 dimers through treatment with butyrate enhanced their activity through tetrameric formation in CRC cells. This switching caused the downregulation of lactate production and the inhibition of CRC cell proliferation [88]. PKM2 has also been reported as a target for vitamin C treatment, by which it inhibits its phosphorylation at serine 37 residue of PKM2 [79]. Oxymatrine was found to inhibit metastasis and attenuate the aerobic glycolysis in CRC cells, and the inhibition was associated with the blocking of PKM2 activity [3]. These studies provide a novel insight into PKM2′s potential role in inducing CRC metastasis by enhancing the Warburg effect.

#### 3.2.3. Hexokinase 2 (HK2)

The enzyme HK2 mediates the first irreversible step of glycolysis, promoting the phosphorylation of glucose to glucose-6-phosphate. HK2 has been reported to be upregulated in different cancers, including CRC, and its upregulation was linked to poor prognoses [89,90]. HK2 has also been shown to be regulated by different oncogenes and tumor suppresser genes, including *HIF1A*, *c-Myc*, and *TP53* [91]. FOX transcription factor E1 (FOXE1) was recently shown to function as a tumor suppresser in CRC cases via downregulation of HK2-induced aerobic glycolysis [6]. 

With regard to epigenetic regulation, Gregersen et al. reported that HK2 is the main target for miR-143 and that miR-143 loss is significantly associated with increased glycolysis activity in CRC cells [92]. Moreover, lncRNAs (such as MEG3 and KCNQ1OT1) were also reported to regulate HK2 activity [37,57]. Both these lncRNAs play an important role in regulating the Warburg effect in CRC cells. CRC cells overexpressing MEG have shown a reduced expression of HK2 and an inhibition of glycolysis metabolism [57]. On the other hand, KCNQ1OT1 acts as a proteasome inhibitor to increase the stability of HK2, thereby increasing aerobic glycolysis in CRC cells [37]. 

In addition to its tumorigenic activity, HK2 provides CRC cells with an environment that is resistant to chemotherapy. One study recently demonstrated that HK2 promotes B7-H3-induced chemoresistance in CRC cells [89]. B7-H3 is an immunoregulatory protein reported to effectively enhance the Warburg effect in CRC. Remarkably, the inhibition of HK2 by 2-deoxyglucose sensitized CRC cells to both oxaliplatin and 5-FU treatment [89]. 

#### 3.2.4. Glucose Transporter 1 (GLUT1)

GLUT1 is a member of the GLUT transporter family (SLC2) [2] and is the master glucose transporter in almost all mammalian cell types, but with different expression levels. However, cancer cells exhibit elevated expression of *GLUT1*, and this upregulation is significantly correlated with poor prognoses and abnormal metabolism in different cancers, including CRC. One of the hallmarks of increased glycolytic activity is 18F-FDG accumulation in cancer cells [93], and GLUT1 is the main factor causing such 18F-FDG accumulation. Also, *KRAS* and *BRAF* mutations associated with abnormal glycolysis metabolism require the upregulation of *GLUT1* expression [2], and CRC cells with aberrant SMAD4 expression are frequently associated with enhanced GLUT1 activity [24]. *GLUT1* is therefore considered an attractive target with great potential for inhibiting CRC progression by reprogramming cancer cell metabolism. 

Different epigenetic mechanisms have been reported to regulate *GLUT1* expression in CRC cells. Both miR-760 and miRNA-143 bind to *GLUT1* and inhibit its activity [94,95]. miR-760-mediated *GLUT1* inhibition can be restored by the circRNA circDENND4C, which functions as an miRNA sponger to upregulate glycolysis in CRC [94]. On the other hand, miRNA-143 inhibits glucoses uptake and glycolysis in CRC cells by inhibiting *GLUT1* expression [95]. Recently, it was reported that *GLUT1* is subjected to methylation by the m6A regulatory enzyme METTL3, upregulating its expression and facilitating CRC progression by enhancing glucose uptake and lactate production [96]. Overall, these studies provide a better understanding of the mechanisms by which *GLUT1* expression might be deregulated in CRC cells, thereby enabling the identification of novel targets to regulate its expression. 

Inhibition of *GLUT1* expression has been shown to be facilitated by a group of anti-cancer therapies, including DT-13, Oridonin, and Oxymatrine [2,3,97], and by butyrate, which functions as a glycolysis inhibitor in CRC cells [98]. However, it is still unclear whether butyrate affects *GLUT1* expression as an HDAC inhibitor, thus warranting further studies. These studies, as mentioned earlier, may provide insights into the relationship between *GLUT1* inhibition and chemoresistance in CRC cells.

## 4. Conclusions and Future Research

In summary, CRC cells benefit from the Warburg effect’s ability to enhance their bioenergetic balance and obtain growth-related advantages from glycolysis-derived metabolites. Genetic analyses and ncRNA-mediated epigenetic research have provided insights into the molecular mechanisms of genes involved in regulating the Warburg effect and the development of tumorigenesis. In this review, we have presented molecular insights into the clinical impacts of oncogenic alterations and the effects of overexpression of transcription factors (*KRAS*, APC, *c-Myc*, P53, and HIF1-α), metabolite transporters (GLUT1), and glycolytic enzymes (HK2, PKM2, PDK1, and LDH) on the Warburg effect in CRC cells. For the first time, we have summarized recent pieces of literature showing the importance of miRNAs and lncRNAs as epigenetic mediators regulating the Warburg effect in CRC cells (Table 1). Genetic mutations and epigenetic alterations that deregulate transcription factors, metabolic transporters, and glycolytic enzymes have been associated with poor prognoses and may be associated with chemoradiotherapy resistance in CRC patients. Novel small molecules targeting these enzymes or transporters exert significant anti-proliferative effects. Hence, glycolytic enzymes and metabolite transporters may be used as biomarkers for predicting CRC prognoses and crucial therapeutic targets. Previous studies have demonstrated that the inhibition of epigenetic factors impacts cancer cell metabolism, although further studies are required to fully understand the effectiveness of these inhibitors on the underlying mechanisms in CRC cells. Future studies, particularly translational research, should incorporate ncRNA analysis of epigenomic biomarkers, allowing for personalized treatment using epigenetic modulators. Additionally, combining epigenetic and genetic targeting might be a more effective strategy for delaying CRC progression.

## Figures and Tables

**Table 1:** Warburg effect-mediating molecules and their associated epigenetic alteration, resistance to chemotherapy, and tested anti-glycolytic drugs for CRC.

Genes	Function		Epigenetic Alteration		Therapy Resistance	Anti-Glycolysis Therapy
		Molecule	Expression in CRC	Effect on Glycolysis		
*KRAS*	Oncogenic activator of RAS/MAPK	UNC5B-AS1 [50]	Upregulated	Activating	Anti-EGFR [47,48]	3-BrPA [19], ascorbic acid [18]
*c-Myc*	Oncogenic Transcription factor	MEG3 [57] GLCC1 [26] miR-181d [58] miR-124 [55]	Downregulated Upregulated Upregulated Downregulated	Inhibitory Activating Activating Inhibitory	N\A	vitamin D [57] Ketamine [59] Dioscin [60]
*HIF1A*	Hypoxia inducible transcription factor	METTL3 [67] YTHDF1 [67] HIFAL [38]	Upregulated Upregulated Upregulated	Activating Activating Inhibitory	5-FU [66]	Rosmarinic acid [65]
*APC*	Tumor suppressor controlling beta-catenin	N\A		N\A	N\A	DT-13 [2] Metformin [70]
*TP53*	Transcription factor and tumor suppresser	N\A		N\A	N\A	DCA [23] FK866 [72]
*PKM2*	An enzyme of aerobic glycolysis	miR-1 [82] miR-133b [82] miR-124 [83] miR-137 [83] miR-340 [83] miR-206 [84] MEG3 [57] FEZF1-AS1 [85] miR-122 [86]	Downregulated Downregulated Downregulated Downregulated Downregulated Downregulated Downregulated Upregulated Downregulated	Inhibitory Inhibitory Inhibitory Inhibitory Inhibitory Inhibitory Inhibitory Activating Inhibitory	Oxaliplatin [86]	Butyrate [88] vitamin C [79] Oxymatrine [3]
*HK2*	An enzyme of aerobic glycolysis	miR-143 [92] MEG3 [57] KCNQ1OT1 [37]	Downregulated Downregulated Upregulated	Inhibitory Inhibitory Activating	Oxaliplatin [89] 5-FU [89]	N\A
*GLUT1*	Glucose transporter	miR-760 [94] miR-143 [95] circDENND4C [94] METTL3 [96]	Downregulated Downregulated Upregulated Upregulated	Inhibitory Inhibitory Activating Activating	5-FU [98]	DT-13 [2] Oridonin [97] Oxymatrine [3] Butyrate [98]

## Data Availability

Not applicable.
